# A triclinic polymorph of dicadmium divanadate(V)

**DOI:** 10.1107/S1600536813028869

**Published:** 2013-10-26

**Authors:** Ahmed Ould Saleck, Abderrazzak Assani, Mohamed Saadi, Lahcen El Ammari

**Affiliations:** a Laboratoire de Chimie du Solide Appliquée, Faculté des Sciences, Université Mohammed V-Agdal, Avenue Ibn Battouta, BP 1014, Rabat, Morocco

## Abstract

The title compound, Cd_2_V_2_O_7_, was obtained under hydro­thermal conditions. Different from the known monoclinic form, the new polymorph of Cd_2_V_2_O_7_ has triclinic symmetry and is isotypic with Ca_2_V_2_O_7_. The building units of the crystal structure are two Cd^2+^ cations, with coordination numbers of six and seven, and two V atoms with a tetra­hedral and a significantly distorted trigonal–pyramidal coordination environment, respectively. Two VO_5_ pyramids share an edge and each pyramid is connected to one VO_4_ tetra­hedron *via* a corner atom, forming an isolated V_4_O_14_^8−^ anion. These anions are arranged in sheets parallel to (-211) and are linked through the Cd^2+^ cations into a three-dimensional framework structure.

## Related literature

For Ca_2_V_2_O_7_, isotypic with the title compound, see: Trunov *et al.* (1983 ▶). For the structure of the monoclinic polymorph of Cd_2_V_2_O_7_, see: Au & Calvo (1967 ▶). For the thermal stability of the monoclinic polymorph, see: Krasnenko & Rotermel (2010 ▶). For applications of vanadates, see: Jin *et al.* (2013 ▶); Valverde *et al.* (2012 ▶). For bond-valence analysis, see: Brown & Altermatt (1985 ▶).

## Experimental

### 

#### Crystal data

Cd_2_V_2_O_7_
*M* *_r_* = 438.68
Triclinic, 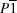

*a* = 6.5974 (2) Å
*b* = 6.8994 (2) Å
*c* = 6.9961 (2) Å
α = 83.325 (1)°
β = 63.898 (1)°
γ = 80.145 (1)°
*V* = 281.45 (1) Å^3^
*Z* = 2
Mo *K*α radiation
μ = 10.65 mm^−1^
*T* = 296 K
0.29 × 0.17 × 0.12 mm

#### Data collection

Bruker X8 APEX diffractometer
Absorption correction: multi-scan (*SADABS*; Bruker, 2009 ▶) *T*_min_ = 0.164, *T*_max_ = 0.376
10113 measured reflections
2134 independent reflections
2077 reflections with *I* > 2σ(*I*)
*R*_int_ = 0.025

#### Refinement

*R*[*F* ^2^ > 2σ(*F* ^2^)] = 0.016
*wR*(*F* ^2^) = 0.037
*S* = 1.21
2134 reflections
100 parameters
Δρ_max_ = 0.70 e Å^−3^
Δρ_min_ = −1.53 e Å^−3^

### 

Data collection: *APEX2* (Bruker, 2009 ▶); cell refinement: *SAINT* (Bruker, 2009 ▶); data reduction: *SAINT*; program(s) used to solve structure: *SHELXS97* (Sheldrick, 2008 ▶); program(s) used to refine structure: *SHELXL97* (Sheldrick, 2008 ▶); molecular graphics: *ORTEP-3 for Windows* (Farrugia, 2012 ▶) and *DIAMOND* (Brandenburg, 2006 ▶); software used to prepare material for publication: *publCIF* (Westrip, 2010 ▶).

## Supplementary Material

Crystal structure: contains datablock(s) I. DOI: 10.1107/S1600536813028869/wm2776sup1.cif

Structure factors: contains datablock(s) I. DOI: 10.1107/S1600536813028869/wm2776Isup2.hkl

Additional supplementary materials:  crystallographic information; 3D view; checkCIF report

## Figures and Tables

**Fig. 1.**
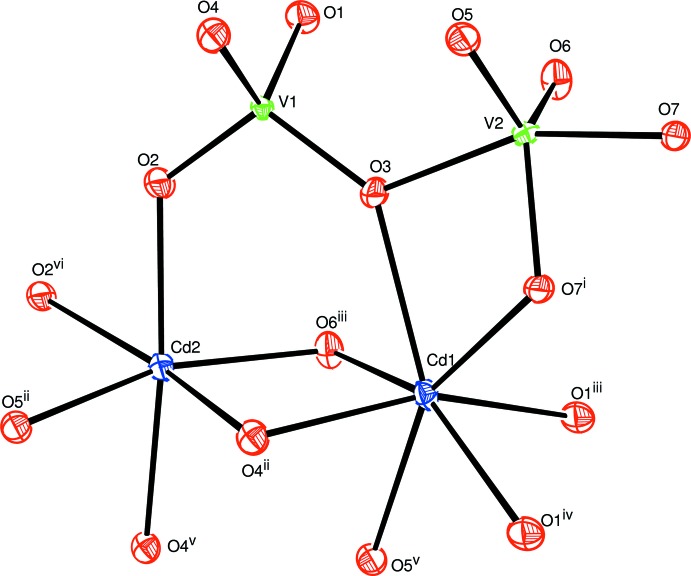
The coordination environment of the Cd and V sites in the crystal structure of the title compound, triclinic Cd2V2O7. Displacement ellipsoids are drawn at the 70% probability level. [Symmetry codes: (i) -x, -y + 1, -z + 1; (ii) -x + 1, -y, -z + 1; (iii) -x + 1, -y + 1, -z + 1; (iv) x - 1, y, z + 1; (v) x, y, z + 1; (vi) -x + 2, -y, -z + 1.]

**Fig. 2.**
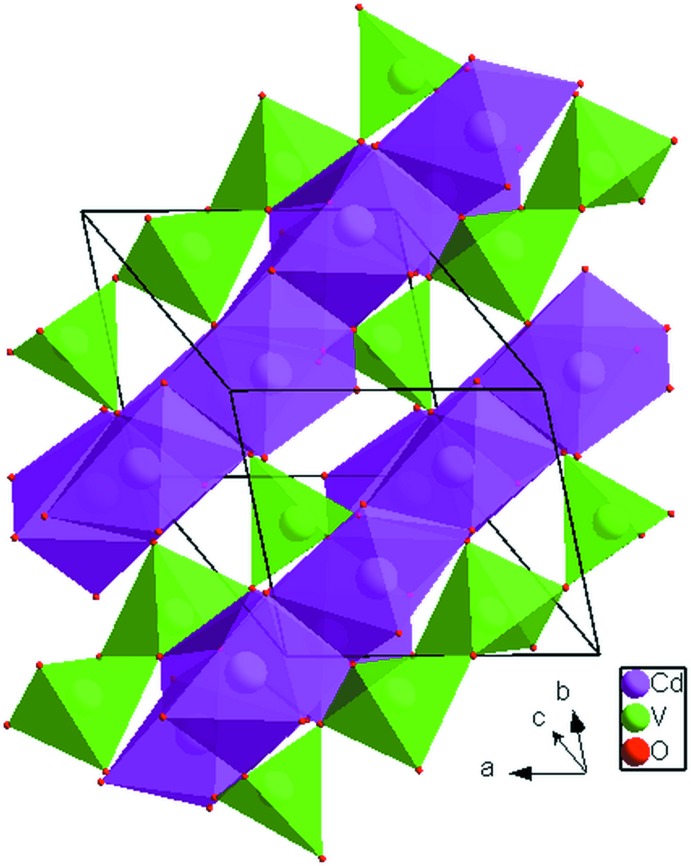
Polyhedral representation of triclinic Cd2V2O7, showing the sheets of cadmium- and vanadium-oxygen polyhedra.

**Fig. 3.**
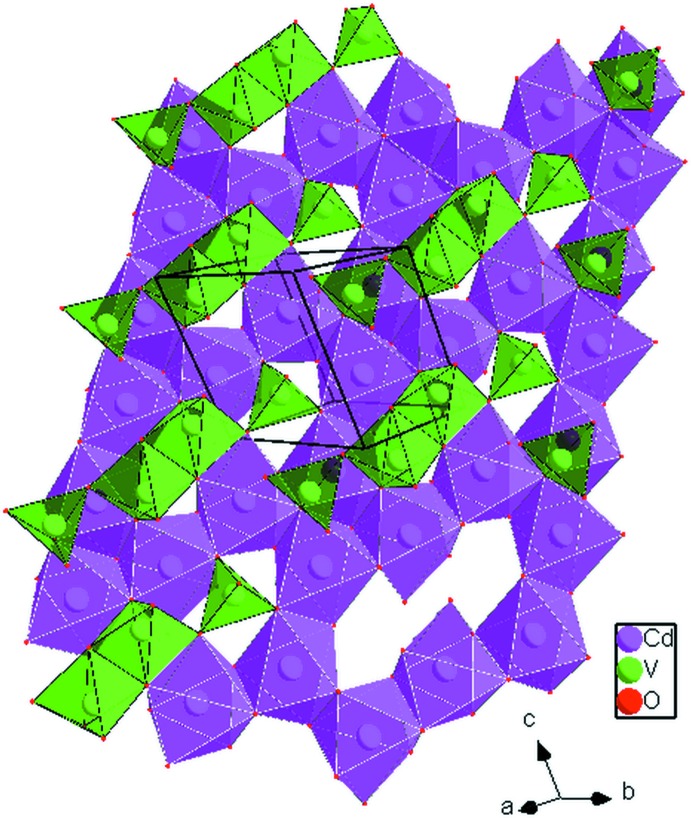
A three dimensional view of the crystal structure of triclinic Cd2V2O7 showing the stacking of the cadium and vanadium layers that extend parallel to (211).

## supplementary crystallographic information

### 1. Comment

Vanadate-based compounds have received a great extent of interest and still remain promising functional materials. Their structural diversity, mainly associated with the ability of vanadium to form different anions like (VO_4_)^3-^, (V_2_O_7_)^4-^, (V_4_O_12_)^4-^, (V_10_O_28_)^6-^, is strongly required for catalysis applications (Jin *et al.*, 2013; Valverde *et al.*, 2012).

A bibliographic analysis revealed that pyrovanadates with the (V_2_O_7_)^4-^ anion can adopt different symmetries. For example, Cu_2_V_2_O_7_, Mn_2_V_2_O_7_ and Mg_2_V_2_O_7_ are polymorphic and exhibit both monoclinic and triclinic varieties, the Cu-member additionally an orthorhombic form. In the case of Co_2_V_2_O_7_, Ni_2_V_2_O_7_, and Cd_2_V_2_O_7_ only one monoclinic form is yet known, for Zn_2_V_2_O_7_ two monoclinic forms are reported. Ca_2_V_2_O_7_ and Ba_2_V_2_O_7_ crystallize in the triclinic system, Sr_2_V_2_O_7_ is likewise polymorphic, with triclinic and tetragonal varieties. We report here on the crystal structure determination of a new form of Cd_2_V_2_O_7_ that was hydrothermally synthesized. In contrast to the known monoclinic polymorph (Au & Calvo, 1967) that is stable from ambient temperature to 1173 K (Krasnenko *et al.*, 2010), this new form has triclinic symmetry and is isotypic with Ca_2_V_2_O_7_ (Trunov *et al.*, 1983).

The structure of the title compound is built up from two types of vanadium sites and two types of cadmium sites, each with a different oxygen coordination as shown in Fig. 1. The coordination environment of V1 is tetrahedral with V1–O distances in the range 1.6882 (13) Å - 1.7708 (13) Å. V2 is surrounded by five oxygen atoms with four V2—O distances ranging from 1.6612 (14) Å to 1.8535 (13) Å and the fifth O atom at a longer distance V2–O1 = 2.0348 (13) Å, forming a distorted trigonal V2O_5_^5-^ pyramid. The bond valence sum calculation (Brown & Altermatt, 1985) for V1 and V2 are as expected, *viz*. 4.99 and 5.11 valence units, respectively. This result confirms the involvement of O1 in the V2 environment. The V2O_5_^5-^ pyramids build a dimeric unit (V2)_2_O_8_^6-^ by sharing an edge. A corner atom (O1) of each of the pyramids is also part of a V1O_4_ tetrahedron. This linkeage leads to the formation of a centrosymmetric (V_4_O_14_)^8-^ anion. This type of anion is rarely encountered in the crystal chemistry of pyrovanadates. Like in the monoclinic Cd_2_V_2_O_7_ variety (Au and Calvo, 1967), (V_2_O_7_)^4-^ groups made up from two corner-sharing VO_4_ tetrahedra are more commonly observed.

The two independent cadmium sites Cd1 and Cd2 are surrounded by six and seven oxygen atoms, respectively, in the form of distorted polyhedra. The Cd—O distance range from 2.2401 (13) Å to 2.5300 (13) Å for Cd1O_7_ and from 2.2449 (14) Å to 2.4562 (14) Å for Cd2O_6_. As shown in Fig. 2, edge-sharing CdO_6_ and CdO_7_ polyhedra built up sheets parallel to (211) with 8-membered open rings. Two adjacent layers are linked by the (V_4_O_14_)^8-^ groups into a three-dimensional framework (Fig. 3). In the monoclinic Cd_2_V_2_O_7_ polymorph, the CdO_6_ octahedra share edges and form sheets with six-membered rings that are linked by (V_2_O_7_)^4-^ groups.

### 2. Experimental

Crystals of the title compound were isolated from the hydrothermal treatment of cadmium oxide, ammonium metavanadate and zinc oxide in a molar ratio Cd:V:Zn = 11:8:1. Zinc oxide, not present in the obtained crystals, presumably acted as a mineralizing agent. The hydrothermal reaction was conducted in a 23 ml Teflon-lined autoclave, filled to 50% with distilled water and under autogeneous pressure at 493 K for four days. After being filtered off, washed with deionized water and air dried, the reaction product consists of colourless sheet-shaped crystals corresponding to the title compound.

### 3. Refinement

The highest peak and the deepest hole in the final Fourier map are at 1.69 Å and 0.08 Å, from O2 and Cd2, respectively. Reflections (200) and (020) were omitted from the refinement due to large differences between observed and calculated intensities.

### Crystal data

**Table:**

Cd_2_V_2_O_7_	*Z* = 2
*M**_r_* = 438.68	*F*(000) = 396
Triclinic, *P*1	*D*_x_ = 5.176 Mg m^−^^3^
Hall symbol: -P 1	Mo *K*α radiation, λ = 0.71073 Å
*a* = 6.5974 (2) Å	Cell parameters from 2717 reflections
*b* = 6.8994 (2) Å	θ = 3.0–33.1°
*c* = 6.9961 (2) Å	µ = 10.65 mm^−^^1^
α = 83.325 (1)°	*T* = 296 K
β = 63.898 (1)°	Block, colourless
γ = 80.145 (1)°	0.29 × 0.17 × 0.12 mm
*V* = 281.45 (1) Å^3^	

### Data collection

**Table:**

Bruker X8 APEX diffractometer	2134 independent reflections
Radiation source: fine-focus sealed tube	2077 reflections with *I* > 2σ(*I*)
Graphite monochromator	*R*_int_ = 0.025
φ and ω scans	θ_max_ = 33.1°, θ_min_ = 3.0°
Absorption correction: multi-scan (*SADABS*; Bruker, 2009)	*h* = −10→10
*T*_min_ = 0.164, *T*_max_ = 0.376	*k* = −10→10
10113 measured reflections	*l* = −10→10

### Refinement

**Table:**

Refinement on *F*^2^	0 restraints
Least-squares matrix: full	Primary atom site location: structure-invariant direct methods
*R*[*F*^2^ > 2σ(*F*^2^)] = 0.016	Secondary atom site location: difference Fourier map
*wR*(*F*^2^) = 0.037	*w* = 1/[σ^2^(*F*_o_^2^) + (0.0148*P*)^2^ + 0.2572*P*] where *P* = (*F*_o_^2^ + 2*F*_c_^2^)/3
*S* = 1.21	(Δ/σ)_max_ = 0.001
2134 reflections	Δρ_max_ = 0.70 e Å^−^^3^
100 parameters	Δρ_min_ = −1.53 e Å^−^^3^

### Special details

**Table:**

Geometry. All e.s.d.'s (except the e.s.d. in the dihedral angle between two l.s. planes) are estimated using the full covariance matrix. The cell e.s.d.'s are taken into account individually in the estimation of e.s.d.'s in distances, angles and torsion angles; correlations between e.s.d.'s in cell parameters are only used when they are defined by crystal symmetry. An approximate (isotropic) treatment of cell e.s.d.'s is used for estimating e.s.d.'s involving l.s. planes.
Refinement. Refinement of *F*^2^ against all reflections. The weighted *R*-factor *wR* and goodness of fit *S* are based on *F*^2^, conventional *R*-factors *R* are based on *F*, with *F* set to zero for negative *F*^2^. The threshold expression of *F*^2^ > 2σ(*F*^2^) is used only for calculating *R*-factors(gt) *etc*. and is not relevant to the choice of reflections for refinement. *R*-factors based on *F*^2^ are statistically about twice as large as those based on *F*, and *R*- factors based on all data will be even larger.

### Fractional atomic coordinates and isotropic or equivalent isotropic displacement parameters (Å^2^)

**Table:**

	*x*	*y*	*z*	*U*_iso_*/*U*_eq_	
Cd1	0.24214 (2)	0.336697 (18)	0.83258 (2)	0.00767 (4)	
Cd2	0.74980 (2)	0.034380 (18)	0.75748 (2)	0.00845 (4)	
V1	0.71038 (5)	0.16450 (4)	0.25864 (4)	0.00469 (5)	
V2	0.22836 (5)	0.45517 (4)	0.34409 (5)	0.00542 (5)	
O1	0.8612 (2)	0.3328 (2)	0.0816 (2)	0.0100 (2)	
O2	0.8622 (2)	0.0439 (2)	0.3907 (2)	0.0099 (2)	
O3	0.4592 (2)	0.2893 (2)	0.4363 (2)	0.0092 (2)	
O4	0.6546 (2)	−0.00638 (19)	0.1233 (2)	0.0086 (2)	
O5	0.2714 (2)	0.29481 (19)	0.1660 (2)	0.0093 (2)	
O6	0.3839 (2)	0.6438 (2)	0.2436 (2)	0.0104 (2)	
O7	−0.0496 (2)	0.5892 (2)	0.3678 (2)	0.0100 (2)	

### Atomic displacement parameters (Å^2^)

**Table:**

	*U*^11^	*U*^22^	*U*^33^	*U*^12^	*U*^13^	*U*^23^
Cd1	0.00672 (6)	0.00774 (6)	0.00900 (6)	−0.00006 (4)	−0.00431 (4)	0.00047 (4)
Cd2	0.00779 (6)	0.00795 (6)	0.00808 (6)	0.00031 (4)	−0.00277 (4)	0.00069 (4)
V1	0.00423 (11)	0.00515 (11)	0.00483 (11)	−0.00033 (8)	−0.00222 (9)	−0.00007 (9)
V2	0.00472 (11)	0.00635 (12)	0.00446 (11)	−0.00089 (9)	−0.00115 (9)	−0.00065 (9)
O1	0.0089 (5)	0.0096 (5)	0.0098 (5)	−0.0031 (4)	−0.0025 (5)	0.0023 (4)
O2	0.0080 (5)	0.0124 (6)	0.0090 (5)	0.0009 (4)	−0.0044 (4)	0.0003 (4)
O3	0.0070 (5)	0.0116 (6)	0.0079 (5)	0.0021 (4)	−0.0032 (4)	−0.0013 (4)
O4	0.0110 (6)	0.0068 (5)	0.0095 (5)	−0.0012 (4)	−0.0054 (5)	−0.0014 (4)
O5	0.0128 (6)	0.0079 (5)	0.0083 (5)	0.0002 (4)	−0.0057 (5)	−0.0015 (4)
O6	0.0081 (5)	0.0079 (5)	0.0147 (6)	−0.0021 (4)	−0.0046 (5)	0.0014 (4)
O7	0.0056 (5)	0.0157 (6)	0.0064 (5)	0.0026 (4)	−0.0020 (4)	0.0004 (4)

### Geometric parameters (Å, º)

**Table:**

Cd1—O7^i^	2.2401 (13)	Cd2—O4^ii^	2.4562 (14)
Cd1—O4^ii^	2.2898 (13)	V1—O1	1.6882 (13)
Cd1—O6^iii^	2.3083 (14)	V1—O2	1.7028 (14)
Cd1—O1^iii^	2.3345 (14)	V1—O3	1.7265 (13)
Cd1—O1^iv^	2.3476 (13)	V1—O4	1.7708 (13)
Cd1—O5^v^	2.4043 (13)	V2—O5	1.6612 (14)
Cd1—O3	2.5300 (13)	V2—O6	1.6885 (14)
Cd2—O6^iii^	2.2449 (14)	V2—O7	1.8530 (13)
Cd2—O5^ii^	2.2858 (13)	V2—O7^i^	1.8535 (13)
Cd2—O2^vi^	2.2894 (14)	V2—O3	2.0348 (13)
Cd2—O2	2.3327 (14)	V2—V2^i^	2.8482 (6)
Cd2—O4^v^	2.3459 (13)		
			
O7^i^—Cd1—O4^ii^	114.29 (5)	O2^vi^—Cd2—O2	74.71 (5)
O7^i^—Cd1—O6^iii^	131.24 (5)	O6^iii^—Cd2—O4^v^	96.72 (5)
O4^ii^—Cd1—O6^iii^	83.66 (5)	O5^ii^—Cd2—O4^v^	75.42 (5)
O7^i^—Cd1—O1^iii^	85.81 (5)	O2^vi^—Cd2—O4^v^	102.17 (5)
O4^ii^—Cd1—O1^iii^	157.35 (5)	O2—Cd2—O4^v^	174.49 (5)
O6^iii^—Cd1—O1^iii^	90.73 (5)	O6^iii^—Cd2—O4^ii^	81.30 (5)
O7^i^—Cd1—O1^iv^	76.73 (5)	O5^ii^—Cd2—O4^ii^	75.69 (5)
O4^ii^—Cd1—O1^iv^	94.46 (5)	O2^vi^—Cd2—O4^ii^	160.52 (5)
O6^iii^—Cd1—O1^iv^	149.98 (5)	O2—Cd2—O4^ii^	98.40 (5)
O1^iii^—Cd1—O1^iv^	79.55 (5)	O4^v^—Cd2—O4^ii^	83.09 (5)
O7^i^—Cd1—O5^v^	153.49 (5)	O1—V1—O2	109.38 (7)
O4^ii^—Cd1—O5^v^	74.22 (5)	O1—V1—O3	107.57 (7)
O6^iii^—Cd1—O5^v^	73.06 (5)	O2—V1—O3	109.85 (7)
O1^iii^—Cd1—O5^v^	83.15 (5)	O1—V1—O4	109.71 (7)
O1^iv^—Cd1—O5^v^	77.60 (5)	O2—V1—O4	109.65 (7)
O7^i^—Cd1—O3	62.10 (5)	O3—V1—O4	110.65 (6)
O4^ii^—Cd1—O3	86.49 (5)	O5—V2—O6	114.25 (7)
O6^iii^—Cd1—O3	75.19 (5)	O5—V2—O7	99.11 (7)
O1^iii^—Cd1—O3	113.31 (5)	O6—V2—O7	98.25 (7)
O1^iv^—Cd1—O3	134.73 (5)	O5—V2—O7^i^	121.64 (7)
O5^v^—Cd1—O3	144.28 (4)	O6—V2—O7^i^	123.74 (7)
O6^iii^—Cd2—O5^ii^	156.37 (5)	O7—V2—O7^i^	79.57 (6)
O6^iii^—Cd2—O2^vi^	116.20 (5)	O5—V2—O3	92.06 (6)
O5^ii^—Cd2—O2^vi^	87.38 (5)	O6—V2—O3	93.85 (6)
O6^iii^—Cd2—O2	88.75 (5)	O7—V2—O3	158.40 (6)
O5^ii^—Cd2—O2	99.75 (5)	O7^i^—V2—O3	78.83 (6)

Symmetry codes: (i) −*x*, −*y*+1, −*z*+1; (ii) −*x*+1, −*y*, −*z*+1; (iii) −*x*+1, −*y*+1, −*z*+1; (iv) *x*−1, *y*, *z*+1; (v) *x*, *y*, *z*+1; (vi) −*x*+2, −*y*, −*z*+1.
